# Transcriptomic Identification and Functional Assessment of Candidate Host Factors Affecting Akabane Virus Replication

**DOI:** 10.3390/vetsci13101015

**Published:** 2026-09-25

**Authors:** Xiangxiang Tan, Miaomiao Zhang, Canyuan Li, Yuanhang Zhang, Ping Li, Han Gao, Limei Qin, Xiangwei Wang, Shasha Wang, Shengfeng Chen, Zhongxian Chen, Xiaoxia Liang, Dajun Zhang, Keshan Zhang

**Affiliations:** 1 School of Animal Science and Technology, Foshan University, Foshan 528231, China; 13030275112@163.com (X.T.); 13526707484@163.com (M.Z.); liyuan9954@163.com (C.L.); zyhh105@163.com (Y.Z.); liping00716@163.com (P.L.); gh1@fosu.edu.cn (H.G.); qlm@fosu.edu.cn (L.Q.); wangxiangwei@fosu.edu.cn (X.W.); wangshasha@fosu.edu.cn (S.W.); chenshengfeng@fosu.edu.cn (S.C.); 2 Nanhai District Bureau of Agriculture and Rural Affairs, Foshan 528200, China; czseen@163.com (Z.C.); xt0219@126.com (X.L.)

**Keywords:** AKAV, RNA sequencing, innate immune response, antiviral factors

## Abstract

Akabane virus (AKAV) causes abortion, stillbirth, and congenital malformations in ruminants, yet the transcriptional response of Madin–Darby bovine kidney (MDBK) cells and the roles of infection-responsive host genes in AKAV replication remain incompletely characterized. We profiled AKAV-infected and time-matched mock-infected MDBK cells by RNA sequencing at 12 and 24 h post-infection (hpi). Differentially expressed genes (DEGs) were examined using Gene Ontology functional classification and Kyoto Encyclopedia of Genes and Genomes pathway enrichment analysis, six DEGs were evaluated by RT-qPCR, and *OAS2* and *ZBP1* were assessed by overexpression in BHK-21 cells. We identified 119 DEGs at 12 hpi (82 upregulated and 37 downregulated) and 149 at 24 hpi (85 upregulated and 64 downregulated). The DEG sets were associated with antiviral defense, interferon responses, pattern recognition receptor signaling, DNA replication, cell cycle regulation, chromosome organization, and metabolism. RT-qPCR measurements were consistent with the RNA-seq trends. *OAS2* and *ZBP1* transcripts were significantly upregulated at 12 hpi, while *ZBP1* transcript levels remained significantly elevated at 24 hpi. Overexpression of either gene was associated with reductions in AKAV Gc protein abundance, viral RNA levels, and infectious titers. These results define transcriptional changes in AKAV-infected MDBK cells and nominate *OAS2* and *ZBP1* as candidate host factors for further investigation, while their endogenous roles in bovine cells remain to be established.

## Simple Summary

Akabane virus can cause abortion, stillbirth, and malformed offspring in cattle, sheep, and goats. These outcomes can cause substantial losses for livestock producers. Earlier work examined gene activity in cattle joint lining cells, supporting cells from cattle testes, and sheep kidney cells, leaving responses in cattle kidney cells less well described. We examined infected cattle kidney cells at 12 and 24 h and compared them with uninfected cells sampled at the same times. Infection changed the activity of 119 genes at 12 h and 149 genes at 24 h. Many were involved in antiviral defense, detection of infection, cell division, maintenance of genetic material, and energy use. A laboratory test confirmed changes in six genes. Two strongly responsive genes were examined more closely because their proteins participate in antiviral defense. Increasing production of either protein in hamster kidney cells reduced the amounts of viral protein, viral genetic material, and infectious virus. Their natural effects in cattle kidney cells remain to be established. The results broaden current knowledge of cell responses to Akabane virus and may support future efforts to protect animal health and reduce livestock losses.

## 1. Introduction

Akabane virus (AKAV) is transmitted by arthropods and belongs to the genus *Orthobunyavirus* in the family *Peribunyaviridae* [1]. The virus mainly infects cattle and sheep and has been reported across Asia, Africa, the Middle East, and Oceania [2,3,4,5,6]. Infection in adult animals is often clinically inapparent. Infection during pregnancy can cause abortion, stillbirth, premature delivery, arthrogryposis, and hydranencephaly, with substantial consequences for ruminant reproduction [7,8,9].

AKAV has a segmented, negative-sense RNA genome and relies on host protein synthesis, cellular membranes, and metabolic resources for replication [1,10]. RNA species produced during replication can be detected by pattern recognition receptors, leading to interferon production and the expression of interferon-stimulated genes [11,12,13,14]. Viral infection can also disturb cellular transcription, metabolism, cell cycle progression, and stress responses [15,16,17,18]. The origin of these changes is not always clear. Some form part of the host response, while others arise from viral replication or cellular injury. Examining the transcriptome at defined stages of infection allows antiviral gene expression to be considered alongside changes in other cellular processes.

Most AKAV studies have addressed epidemiology, genetic variation, clinical pathology, or cell entry [9,10,19]. Recent transcriptomic studies have begun to characterize host responses to AKAV infection, revealing transcriptional changes in primary bovine cells and Madin–Darby ovine kidney (MDOK) cells and identifying *Rnd1* as an antiviral factor associated with tumor necrosis factor alpha (TNF-α) signaling [20]. However, studies characterizing the early transcriptional response of Madin–Darby bovine kidney (MDBK) cells to AKAV infection remain relatively limited, and the potential relationship between infection-responsive host genes identified in this cell system and AKAV replication has not been fully explored. RNA-seq can define infection-associated expression patterns, while further functional assessment can help clarify whether selected host genes are associated with changes in viral replication [21,22].

In this study, we compared AKAV-infected MDBK cells with mock-infected cultures collected at 12 and 24 hpi. RNA-seq data were examined by Gene Ontology (GO) functional classification and Kyoto Encyclopedia of Genes and Genomes (KEGG) pathway enrichment analysis, and selected expression changes were assessed by quantitative reverse transcription PCR. *OAS2* and *ZBP1* were selected for functional assessment from genes significantly upregulated at 12 hpi because both have established antiviral annotations. *ZBP1* also remained significantly upregulated at 24 hpi. Their effects on AKAV replication were tested by plasmid overexpression in BHK-21 cells, followed by measurements of Gc protein abundance, viral RNA, and infectious virus titers.

## 2. Materials and Methods

### 2.1. Cell Culture and Sample Preparation

MDBK and baby hamster kidney-21 (BHK-21) cells were obtained from stocks maintained in our laboratory and cultured in high-glucose DMEM (Gibco, Grand Island, NY, USA) supplemented with 10% fetal bovine serum (Gibco, Grand Island, NY, USA) and 1% penicillin–streptomycin solution (Solarbio, Beijing, China). Cultures were kept at 37 °C in a humidified incubator with 5% CO_2_. AKAV strain FS202301 was isolated in our laboratory and propagated in MDBK cells. The viral stock had an infectivity titer of 10^6.5^ TCID_50_ per 0.1 mL, determined by endpoint dilution and calculated using the Reed–Muench method. Its genome sequences are available under GenBank accession numbers PQ567126, PQ567127, and PQ567128.

RNA-seq samples were prepared from MDBK cultures at 80% to 90% confluence. Cells were inoculated with AKAV at a multiplicity of infection (MOI) of 1. The amount of virus required to achieve the indicated MOI was calculated based on the infectious titer of the virus stock and the number of cells. Virus adsorption was allowed to proceed for 1 h at 37 °C. The inoculum was subsequently removed and replaced with infection medium containing 2% fetal bovine serum. Parallel mock cultures received medium without virus.

### 2.2. RNA Extraction, Library Construction, and Sequencing

Total RNA was isolated from each MDBK cell sample with Trizol reagent (White Shark Biotechnology, Anhui, Bengbu, China) according to the manufacturer’s instructions. RNA yield, purity, and integrity were assessed using an Agilent 2100 Bioanalyzer (Agilent Technologies, Singapore) and RNase-free agarose gel electrophoresis. RNA samples that passed the quality checks were used for library construction.

RNA-seq libraries were prepared using the Hieff NGS^®^ Ultima Dual-mode mRNA Library Prep Kit (Yeasen Biotechnology, Shanghai, China; Cat#12309ES). Poly(A)+ mRNA was enriched using the oligo(dT)-based mRNA Capture Beads supplied with the kit and fragmented at 94 °C for 5 min. First-strand cDNA was synthesized using Strand Specificity Reagent and 1st Strand Enzyme Mix. Second-strand cDNA synthesis, end repair, and A-tailing were performed using 2nd Strand Buffer and 2nd Strand Enzyme Master Mix. Illumina-compatible sequencing adapters (Yeasen Biotechnology, Shanghai, China) were ligated using Ligation Enhancer and Novel T4 DNA Ligase.

Adapter-ligated products were purified and size-selected using Hieff NGS^®^ DNA Selection Beads (Yeasen Biotechnology, Shanghai, China; Cat#12601). Library amplification was performed using 2× Super Canace^®^ II High-Fidelity Mix (Yeasen Biotechnology, Shanghai, China), together with Universal Primer/i5 Primer and Index Primer/i7 Primer. The PCR program consisted of an initial denaturation at 98 °C for 1 min, followed by 12 cycles of 98 °C for 10 s and 60 °C for 75 s, followed by 72 °C for 30 s and a final extension at 72 °C for 5 min.

Following amplification, the libraries were purified using Hieff NGS^®^ DNA Selection Beads and evaluated for library concentration and fragment-size distribution using an Agilent 2100 Bioanalyzer (Agilent Technologies). Qualified libraries were sequenced by Gene Denovo Biotechnology Co., Ltd. (Guangzhou, China) on an Illumina NovaSeq X Plus platform using paired-end 150 bp sequencing.

### 2.3. Transcriptome Data Processing and Gene Expression Quantification

Raw RNA sequencing reads were processed using fastp v0.18.0 to remove adapter sequences and reads that failed the quality filters. The resulting clean reads were aligned to the *Bos taurus* ARS-UCD1.2 reference genome (INSDC accession GCA_002263795.2) and the corresponding Ensembl release 110 gene annotation using HISAT2 v2.1.0. Reference-guided transcript assembly was performed using StringTie v1.3.4, and gene expression was quantified using RSEM v1.2.19. RSEM generated gene-level read counts and transcripts per million (TPM) values. Read counts were retained for differential expression analysis, whereas TPM values were used only for descriptive expression summaries and visualization.

### 2.4. Differential Expression Analysis, GO Functional Classification, and KEGG Pathway Enrichment Analysis

Gene-level read counts were analyzed using DESeq v1.20.0 with default settings. Pairwise comparisons were performed between AKAV-infected cells and the corresponding mock-infected controls at 12 and 24 hpi. Genes with a false discovery rate (FDR) below 0.05 and an absolute log2 fold change greater than 1 were considered differentially expressed genes (DEGs). The same differential-expression criteria were applied to both the 12 and 24 hpi comparisons. For GO functional classification, DEGs were mapped to Gene Ontology annotations and assigned to level 2 categories within the biological process, cellular component, and molecular function domains. Upregulated and downregulated genes assigned to each category were counted separately. The GO classification was descriptive and was not evaluated using an enrichment significance threshold. KEGG pathway enrichment analysis was conducted using KEGG Release 101. Enrichment was assessed against the corresponding KEGG-annotated background gene set using a hypergeometric test, and the resulting *p* values were adjusted using the FDR method. Pathways with *Q* values below 0.05 were considered significantly enriched.

### 2.5. RT-qPCR Validation of RNA-seq Results

To validate the RNA-seq data, six representative DEGs were selected for RT-qPCR analysis. Total RNA was extracted from independent biological samples using Trizol reagent (White Shark Biotechnology, Anhui, Bengbu, China) and reverse-transcribed into cDNA using the SweScript All-in-One RT SuperMix for qPCR Kit (Servicebio, Wuhan, China). Gene-specific primers were designed using NCBI Primer-BLAST (Primer3 v1.1.0; BLAST+ 2.16.0; National Center for Biotechnology Information, Bethesda, MD, USA) based on *Bos taurus* RefSeq transcript sequences. Primer specificity was checked against the *Bos taurus* RefSeq mRNA database. The primers were synthesized by Sangon Biotech Co., Ltd. (Shanghai, China), and their sequences are listed in Table 1. RT-qPCR was performed using 2× RealStar Fast SYBR qPCR Mix (GenStar, Beijing, China) on a QuantStudio 7 Flex Real-Time PCR System (Applied Biosystems, Pittsburgh, PA, USA). *GAPDH* was used as the endogenous control because its transcript abundance showed only limited variation between AKAV-infected and the corresponding time-matched mock-infected samples in the RNA-seq dataset. *GAPDH* did not meet the predefined differential-expression criteria at either 12 or 24 hpi. Relative gene expression levels were calculated using the 2^−ΔΔCt^ method as described by Livak and Schmittgen [23]. Each sample was analyzed with three technical replicates from three independent biological experiments.

### 2.6. Cell Transfection

Expression plasmids based on pcDNA3.1 and encoding FLAG-tagged OAS2 or Myc-tagged ZBP1 were maintained in our laboratory, with the empty pcDNA3.1 vector used as a control. BHK-21 cells were seeded in six-well plates and transfected at approximately 70% confluence with 1, 2, or 3 μg of the indicated expression plasmid using Lipofectamine 3000 Transfection Reagent (Invitrogen, Carlsbad, CA, USA). At 24 h after transfection, the cells were inoculated with AKAV at an MOI of 1 and incubated at 37 °C for 1 h to allow for viral adsorption. The inoculum was removed and replaced with fresh maintenance medium. Cells and culture supernatants were collected at 24 hpi. Cell lysates were used for Western blotting, total cellular RNA was used to quantify AKAV RNA, and culture supernatants were used for virus titration. AKAV-infected cells without plasmid transfection and cells transfected with the empty pcDNA3.1 vector before infection were included as controls.

### 2.7. Western Blot (WB)

Cells were lysed in RIPA buffer (Servicebio, Wuhan, China; catalog no. G2002), and equal amounts of protein were separated by SDS-PAGE and transferred onto 0.45 μm PVDF membranes (MilliporeSigma, Burlington, MA, USA; catalog no. PVH00010). After blocking with 5% bovine serum albumin in TBST, the membranes were incubated overnight at 4 °C with mouse anti-FLAG (FitGene Biotechnology, Guangzhou, China; catalog no. FI01104), anti-Myc (FitGene Biotechnology, Guangzhou, China; catalog no. FI01105), or anti-β-actin antibodies (Proteintech, Wuhan, China; catalog no. 66009-1-Ig). Mouse monoclonal antibodies against the AKAV Gc and N proteins were provided by Dr. Dongjie Chen from the Institute of Animal Inspection and Quarantine, Chinese Academy of Inspection and Quarantine, Beijing, China. After washing with TBST, the membranes were incubated with HRP-conjugated goat anti-mouse IgG (H + L) (Proteintech, Wuhan, China; catalog no. SA00001-1). Protein bands were visualized using a chemiluminescence detection system, with β-actin serving as the loading control.

### 2.8. Indirect Immunofluorescence Assay (IFA)

For indirect immunofluorescence staining, MDBK cell monolayers were fixed in pre-chilled methanol for 1 h. After fixation, the cells were washed with PBS without a separate blocking step. AKAV N protein was detected using a mouse monoclonal anti-AKAV N antibody prepared in our laboratory, diluted 1:200 in PBST and incubated overnight at 4 °C. After washing with PBS, the cells were incubated with Alexa Fluor 488-conjugated goat anti-mouse IgG secondary antibody (Sanying; Tianjin, China; catalog no. SA00013-1), diluted 1:500 in PBST, for 2 h at 37 °C. Cell nuclei were counterstained and mounted using antifade mounting medium containing DAPI (Solarbio, Beijing, China; catalog no. S2110); no separate DAPI staining step was performed. Fluorescence images were acquired using an Axio Vert.A1 microscope (Carl Zeiss, Oberkochen, Germany) equipped with a 20× objective. Brightness and contrast were adjusted uniformly across all images for presentation, without selective modification of individual image regions.

### 2.9. Viral Titration

Virus infectivity in the culture supernatants was measured using BHK-21 cells seeded in 96-well plates. Each sample was prepared as a series of tenfold dilutions in DMEM, and 100 μL of each dilution was added to the cell monolayers. The cultures were examined for cytopathic effects at 12 h intervals over a 72 h period. Endpoint titers were determined by the Reed-Muench method and reported as TCID_50_/0.1 mL.

### 2.10. Statistical Analysis

Statistical calculations were carried out in GraphPad Prism 10.2.1 (GraphPad Software, Boston, MA, USA). Values are reported as the mean ± SD from at least three independent biological replicates, with the number of replicates specified in each figure legend. An unpaired, two-sided Student’s t test was used to compare two independent groups. Data sets containing more than two groups were analyzed by one-way ANOVA with Dunnett’s multiple comparisons test. For the plasmid dose experiments, cells infected with AKAV without plasmid transfection served as the reference group. Cells receiving the empty vector were included to monitor effects associated with transfection and the vector backbone. Experiments examining infection status and sampling time as separate factors were analyzed by two-way ANOVA with Sidak’s multiple comparisons test. *p* values below 0.05 were considered statistically significant.

## 3. Results

### 3.1. Establishment of AKAV Infection in MDBK Cells

MDBK cells were inoculated with AKAV at an MOI of 1 and examined for N protein expression at 0, 12, and 24 hpi. No N-specific fluorescence was visible at 0 hpi. Staining appeared at 12 hpi and increased at 24 hpi (Figure 1A). Consistent with this observation, Western blotting detected no Gc protein signal at 0 hpi, whereas a signal was observed at 12 hpi and became stronger at 24 hpi (Figure 1B). The increasing N and Gc protein signals showed that AKAV infection was detectable in MDBK cells at 12 and 24 hpi, supporting the selection of these time points for subsequent RNA-seq analysis.

### 3.2. Global Transcriptomic Changes in MDBK Cells Following AKAV Infection

RNA sequencing generated 37,473,674 to 47,165,394 clean reads per library. Non-rRNA reads accounted for 99.32% to 99.51% of the clean reads, with Q30 values ranging from 93.71% to 94.87% and GC contents from 47.53% to 48.11%. Of the non-rRNA reads, 96.88% to 97.40% mapped to the *Bos taurus* reference genome, and 94.95% to 95.50% mapped uniquely (Table 2).

Principal component analysis showed a clear separation between AKAV-infected MDBK cells and the corresponding mock-infected controls at both sampling times, while biological replicates from the same condition clustered closely (Figure 2a). Differential expression analysis was performed using the time-matched mock-infected cells as the reference. At 12 hpi, 119 differentially expressed genes were identified, including 82 upregulated and 37 downregulated genes. At 24 hpi, 149 differentially expressed genes were detected, with 85 showing increased expression and 64 showing decreased expression (Figure 2b).

The volcano plots in Figure 2c,d summarize the infection-related comparisons at 12 and 24 hpi, respectively. log2 fold changes were calculated for AKAV-infected cells relative to the corresponding mock-infected controls. At 12 hpi, *OAS2* and *ZBP1* were significantly upregulated, with log2 fold changes of 9.09 and 4.16 and false discovery rate values of 0.015 and 3.57 × 10^−82^, respectively. *ZBP1* remained significantly upregulated at 24 hpi, with a log2 fold change of 3.34 and a false discovery rate of 4.51 × 10^−48^. Complete differential expression statistics are provided in Supplementary Tables S1 and S2.

### 3.3. GO Functional Classification and KEGG Pathway Enrichment of Differentially Expressed Genes

The DEGs identified at 12 and 24 hpi were assigned to GO level 2 categories within the biological process, cellular component, and molecular function domains (Figure 3a,b). In the biological process domain, many DEGs were classified under cellular process, biological regulation, metabolic process, response to stimulus, and immune system process. Cellular anatomical entity and protein-containing complex contained the largest numbers of DEGs in the cellular component domain, whereas binding and catalytic activity were the principal molecular function categories. At 24 hpi, more downregulated genes were assigned to cellular process, metabolic process, and biological regulation than at 12 hpi.

KEGG analysis revealed time-dependent differences in the pathways associated with AKAV-responsive genes (Figure 3c,d). At 12 hpi, the enriched pathways included NOD-like receptor, RIG-I-like receptor, Toll-like receptor, C-type lectin receptor, and cytosolic DNA-sensing signaling. Necroptosis, complement and coagulation cascades, and pathways related to several viral infections were also represented. By 24 hpi, the enrichment profile included prominent signals related to DNA replication and cell cycle regulation, along with NOD-like receptor and RIG-I-like receptor signaling, cellular senescence, phagosome, and several virus-related pathways. Complete differential expression statistics are provided in Supplementary Tables S1 and S2.

### 3.4. Confirmation of Transcriptomic Changes by RT-qPCR

To validate the expression changes identified by RNA-seq, *IFIT1*, *BST2*, *IFNβ1*, *OAS2*, *ZBP1*, and *ARHGEF6* were selected for RT-qPCR analysis (Figure 4). Compared with the time-matched mock controls, most genes associated with antiviral defense and interferon responses were upregulated following AKAV infection, although the magnitude and temporal pattern of induction varied among genes. *OAS2* transcript levels showed particularly strong induction at 12 hpi and remained relatively elevated at 24 hpi, whereas *ZBP1* mRNA levels remained higher than those in the time-matched mock controls at both time points. *IFIT1*, *BST2*, and *IFNβ1* also showed varying degrees of upregulation at one or both post-infection time points. *ARHGEF6* showed a distinct expression pattern, with its transcript level decreasing at 24 hpi. Overall, RT-qPCR confirmed the direction of the RNA-seq expression changes for the selected genes. For *OAS2* at 24 hpi, RT-qPCR detected sustained induction, whereas the RNA-seq comparison did not reach statistical significance, likely reflecting the low read counts observed for this transcript.

### 3.5. Effects of OAS2 and ZBP1 Overexpression on AKAV Replication

To determine whether OAS2 and ZBP1 affect AKAV replication, BHK-21 cells were transfected with increasing amounts of plasmids encoding FLAG-tagged OAS2 or Myc-tagged ZBP1 and subsequently infected with AKAV. Immunoblotting confirmed the expression of both proteins, with band intensities increasing as the amount of transfected plasmid increased. In contrast, the abundance of AKAV Gc protein progressively decreased in cells overexpressing either OAS2 or ZBP1 (Figure 5a,d). RT-qPCR showed that AKAV RNA abundance was lower than that in the empty-vector control and declined further with increasing plasmid amounts, reaching the lowest level at 3 μg (Figure 5b,e). Viral titers in the culture supernatants showed a similar pattern, decreasing as the amount of either expression plasmid increased (Figure 5c,f). Under these experimental conditions, OAS2 or ZBP1 overexpression was associated with reduced AKAV protein accumulation, viral RNA abundance, and infectious virus production.

## 4. Discussion

Reproductive disorders caused by AKAV have been documented extensively, but the cellular responses accompanying viral replication and the host factors associated with changes in AKAV replication remain less well-characterized. In the present study, AKAV infection of MDBK cells was accompanied by transcriptional changes involving innate immune responses, DNA replication, cell cycle progression, and chromosome organization. *OAS2* and *ZBP1* were selected for functional assessment based on their infection-responsive transcript patterns and antiviral annotations. Their overexpression in BHK-21 cells was associated with lower AKAV Gc protein abundance, viral RNA levels, and infectious virus titers. These observations support *OAS2* and *ZBP1* as infection-responsive candidate host factors warranting further investigation, but do not establish whether endogenous *OAS2* or *ZBP1* is required for restriction of AKAV replication.

The transcriptional profile included several interferon-stimulated and innate immune genes, such as *IFIT1*, *BST2*, *IFNβ1*, *OAS2*, and *ZBP1*. GO functional classification placed the DEGs into broad categories that included response to stimulus and immune system process, while KEGG enrichment analysis associated the infection-responsive gene sets with innate immune signaling, DNA replication, and cell cycle regulation. These results describe an antiviral transcriptional signature rather than functional activation of each annotated pathway. Intracellular pattern recognition receptors can detect viral RNA and replication-associated RNA structures, leading to interferon production and ISG expression [12,14,24]. Similar responses have been reported during infection with Schmallenberg virus, Rift Valley fever virus, influenza virus, and vesicular stomatitis virus [25,26,27,28]. Chen et al. [20] recently described transcriptional changes following AKAV infection in primary bovine Sertoli cells, primary bovine synovial cells, and MDOK cells, and identified *Rnd1* as an antiviral factor associated with TNF-α signaling. These observations extend previous transcriptomic findings to MDBK cells during early AKAV infection. AKAV N and Gc protein abundance continued to increase despite the expression of multiple ISGs, indicating that this response did not prevent ongoing viral replication. AKAV NSs has been reported to suppress innate immune related gene expression in some experimental systems [29], but its contribution to the interferon and ISG profile observed in MDBK cells remains to be determined.

An increasing number of genes involved in cell cycle progression, DNA replication, chromosome organization, and metabolism showed reduced expression. These findings indicate coordinated changes in mRNA abundance rather than reduced functional activity of the corresponding pathways. Viral effects on the cell cycle vary with replication strategy. Some DNA viruses alter cell cycle progression to support genome replication, while infection with several RNA viruses has been associated with impaired DNA synthesis, cell cycle arrest, or metabolic remodeling. Changes in cell cycle and stress-related gene expression have also been reported during infection with influenza virus, Japanese encephalitis virus, and members of the order *Bunyavirales* [30,31,32,33]. In AKAV-infected cells, these transcriptional changes were more extensive at 24 hpi and occurred while viral protein abundance was increasing. The present data cannot determine whether they resulted from a host response, viral remodeling of the intracellular environment, or cellular stress accompanying infection. Measurements of cell cycle distribution, DNA synthesis, metabolic activity, and cell viability are required to distinguish among these possibilities.

Overexpression of OAS2 or ZBP1 was associated with dose-related decreases in AKAV RNA, Gc protein, and infectious virus production, but the similar virological phenotype does not imply a common mechanism. OAS2 is induced by interferon and becomes catalytically active after binding double-stranded RNA. The activated enzyme produces 2′,5′-linked oligoadenylates that stimulate RNase L, which cleaves both viral and cellular RNA [34]. This pathway restricts West Nile virus, dengue virus, and influenza virus [35,36,37]. The reduction in AKAV RNA makes an effect limited to virion assembly or release unlikely. However, the present data cannot distinguish direct degradation of AKAV RNA from reduced RNA synthesis or broader changes in cellular RNA metabolism. Ectopic OAS2 protein expression was confirmed in the overexpression assays, but production of 2′,5′-linked oligoadenylates and RNase L activity were not assessed. ZBP1 transcript levels remained significantly elevated at both sampled time points and ZBP1 has a distinct established antiviral function. During influenza A virus infection, its Zα domains recognize viral Z-RNA, and its RHIM motifs recruit RIPK3. Activation of RIPK3 can induce MLKL-mediated necroptosis or caspase-8-dependent apoptosis, reducing the time available for infected cells to produce virus [38,39,40]. Whether AKAV RNA adopts a Z conformation or is recognized by ZBP1 remains unknown, and activation of RIPK3, MLKL, or caspase-8 was not examined in the present study. Because cell viability and regulated cell death were not directly quantified under the present overexpression conditions, the contribution of broader changes in cellular physiology to the reduced viral output cannot be excluded. Similarly, the present data do not establish whether the phenotype associated with OAS2 overexpression involves RNase L-dependent antiviral activity or broader effects on cellular physiology. Future studies using catalytically inactive OAS2, RNase L depletion, Zα- or RHIM-deficient ZBP1, and modulation of RIPK3 signaling could help distinguish these possibilities.

The RNA-seq and RT-qPCR analyses in this study describe changes in transcript abundance and do not establish corresponding changes in endogenous OAS2 or ZBP1 protein abundance or signaling activity. The sequencing samples were obtained from bovine MDBK cells, whereas the overexpression experiments were performed in hamster BHK-21 cells. BHK-21 cells were selected because preliminary attempts to optimize plasmid transfection in MDBK cells using several transfection reagents did not yield sufficiently efficient and reproducible ectopic expression under our experimental conditions. Nevertheless, species and cell-type differences may influence the response to OAS2 or ZBP1 expression and therefore limit direct comparison between the two experimental systems. Moreover, although increasing OAS2 or ZBP1 expression reduced AKAV output under the tested conditions, these experiments do not establish that endogenous OAS2 or ZBP1 is required for restriction of AKAV replication. Further loss-of-function studies, ideally followed by rescue experiments in MDBK cells or primary bovine cells, will be needed to define their endogenous roles.

## 5. Conclusions

AKAV infection altered gene expression in MDBK cells at 12 and 24 hpi, affecting genes related to antiviral defense, interferon responses, cell proliferation, and genome maintenance. *OAS2* and *ZBP1* transcripts were induced early during infection, and overexpression of either protein in BHK-21 cells was associated with reduced viral Gc protein abundance, RNA levels, and infectious titers. These findings support further investigation of OAS2 and ZBP1 as candidate host factors associated with reduced AKAV output under overexpression conditions; their endogenous roles and underlying mechanisms in bovine cells remain to be determined.

## Figures and Tables

**Figure 1:**
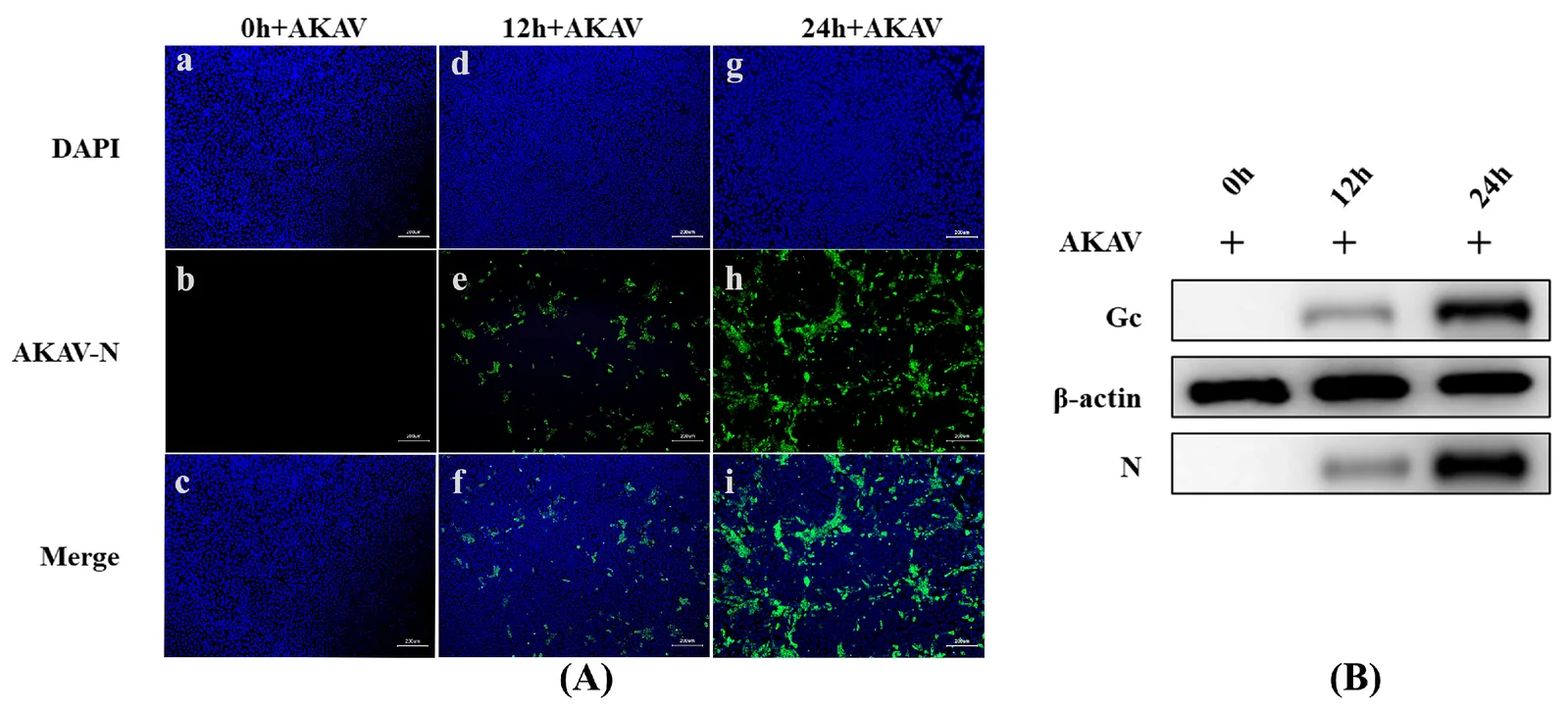
Replication of AKAV in MDBK cells. MDBK cells were infected with AKAV at MOI of 1 and harvested at the indicated time points. Within panel (**A**), images a–c, d–f, and g–i correspond to 0, 12, and 24 hpi, respectively; the upper, middle, and lower rows show DAPI, AKAV-N, and merged signals, respectively. (**B**) WB analysis of AKAV Gc and N protein expression in infected cells at different time points. β-actin was used as the loading control (the original Western blotting pictures can be found in File S1).

**Figure 2:**
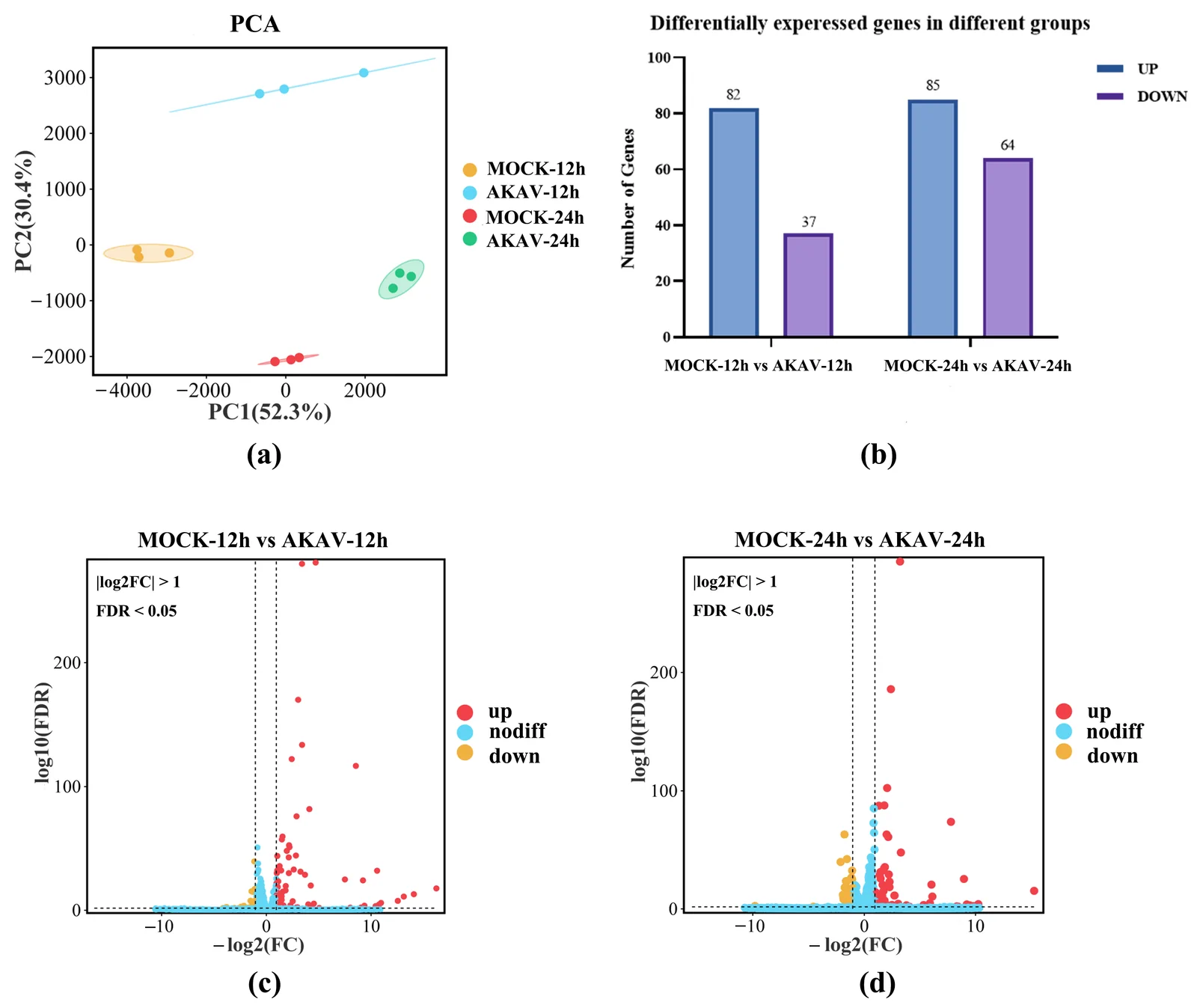
Principal component analysis and differential expression profiles of AKAV-infected MDBK cells. (**a**) Principal component analysis of mock-infected and AKAV-infected MDBK cells collected at 12 and 24 h post-infection. Each point represents one biological replicate. The first and second principal components explain 52.3% and 30.4% of the total variance, respectively. (**b**) Numbers of upregulated and downregulated genes detected in AKAV-infected cells relative to the corresponding mock-infected controls at 12 and 24 h post-infection. The values above the bars indicate the number of genes in each category. (**c**,**d**) Volcano plots for the comparisons between mock-infected and AKAV-infected cells at 12 h and 24 h post-infection, respectively. The x-axis shows the log2 fold change calculated as AKAV-infected relative to mock-infected cells, while the y-axis shows −log10 of the false discovery rate. Red, orange, and blue points indicate significantly upregulated genes, significantly downregulated genes, and genes outside the differential expression thresholds, respectively. Dashed lines indicate the significance thresholds (|log2 fold change| > 1 and FDR < 0.05), which are also labeled directly in panels (**c**,**d**).

**Figure 3:**
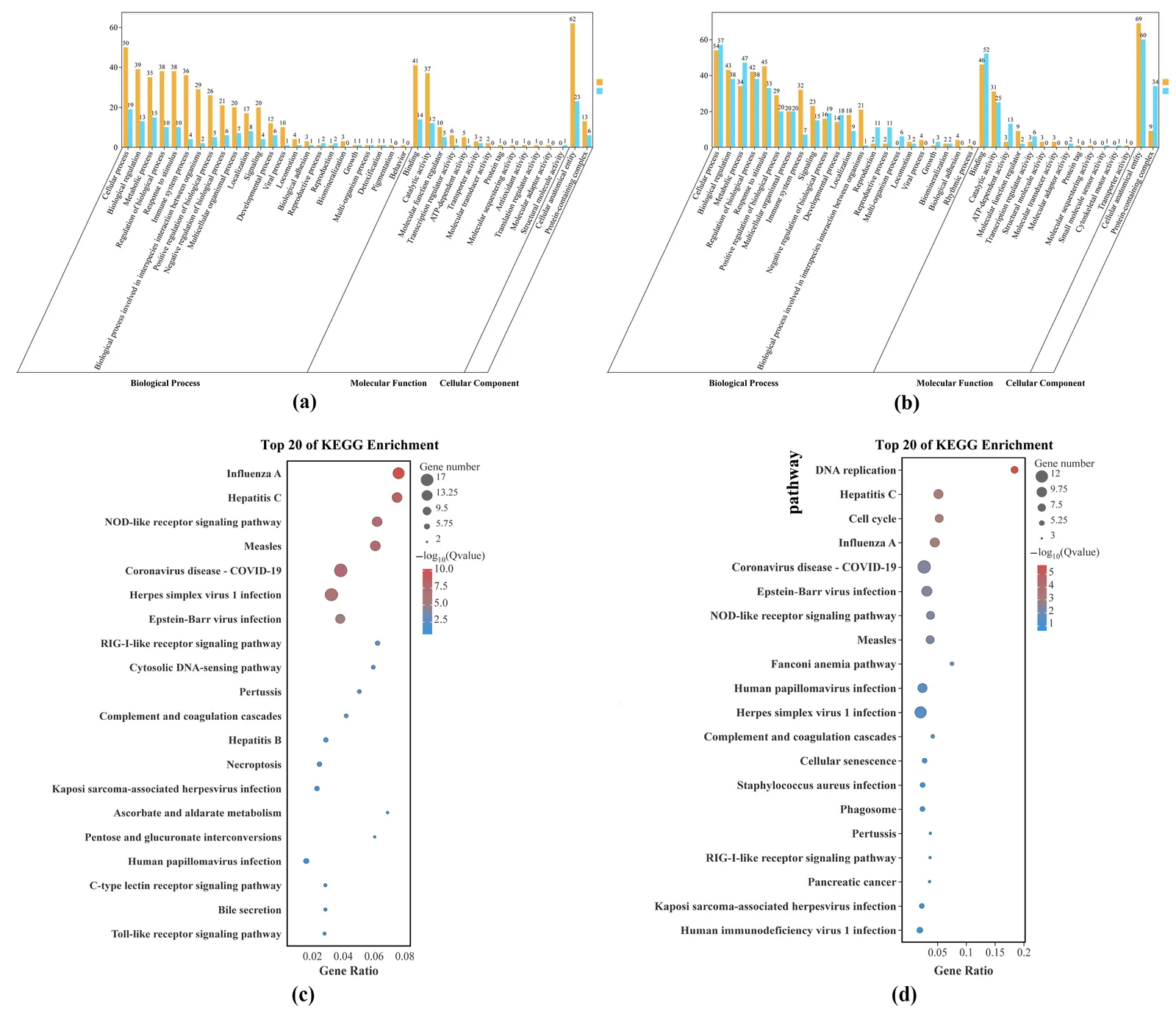
Gene Ontology classification and Kyoto Encyclopedia of Genes and Genomes pathway enrichment of differentially expressed genes in AKAV-infected MDBK cells. (**a**,**b**) Gene Ontology classification of upregulated and downregulated genes identified at 12 and 24 h post-infection, respectively, using the corresponding time-matched mock-infected cells as the reference. Gene Ontology terms are grouped into biological process, cellular component, and molecular function categories. Red and green bars indicate the numbers of upregulated and downregulated genes, respectively. (**c**,**d**) Kyoto Encyclopedia of Genes and Genomes enrichment analysis of differentially expressed genes identified at 12 and 24 h post-infection, respectively. The 20 pathways with the lowest Q values are shown for each comparison. Bubble size represents the number of differentially expressed genes assigned to a pathway, and color indicates −log10(Q value). Pathways with Q < 0.05 were considered significantly enriched.

**Figure 4:**
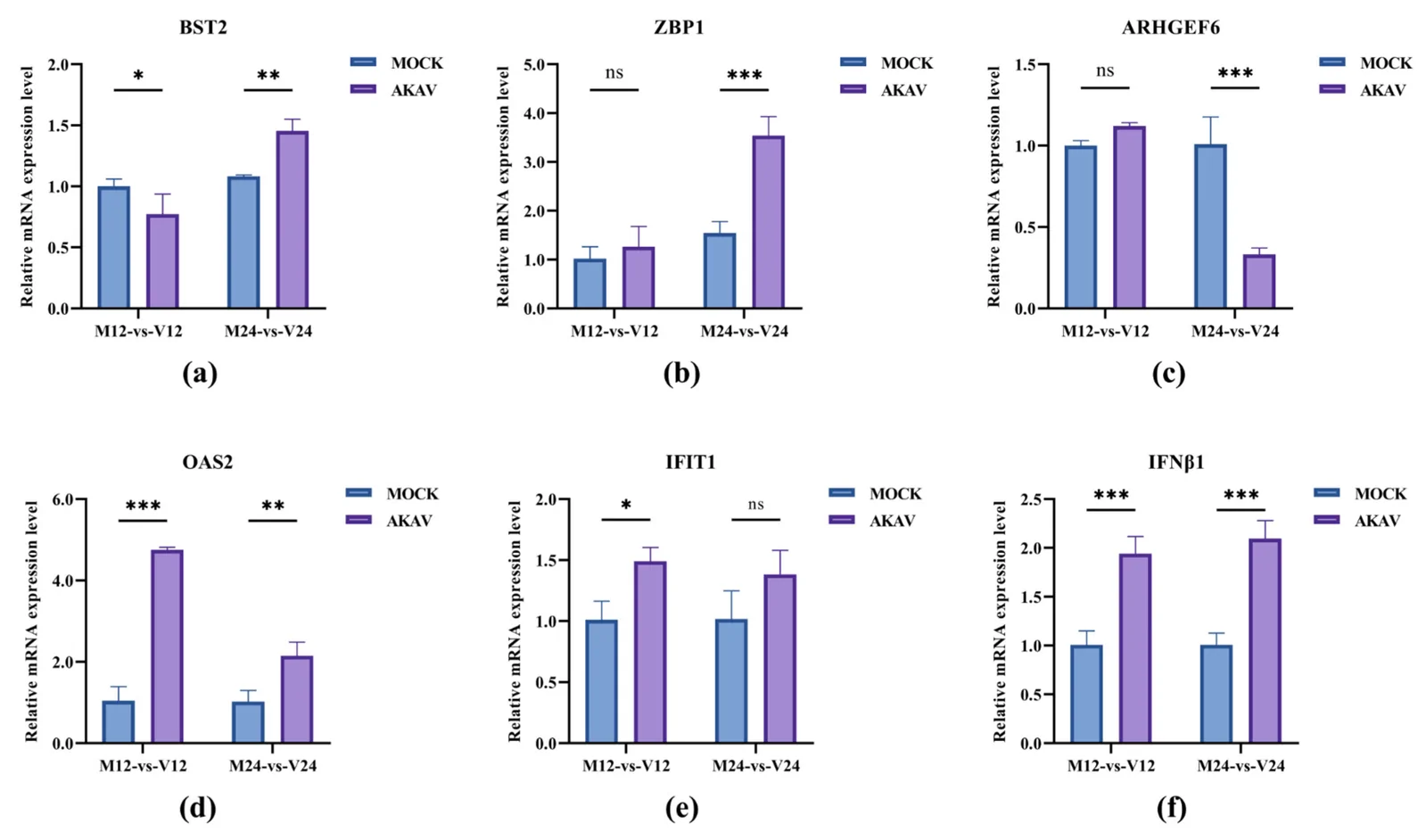
RT-qPCR analysis of selected genes in AKAV-infected MDBK cells. MDBK cells were mock-infected or infected with AKAV, and the relative mRNA levels of *BST2* (**a**), *ZBP1* (**b**), *ARHGEF6* (**c**), *OAS2* (**d**), *IFIT1* (**e**), and *IFNβ1* (**f**) were measured at 12 and 24 hpi. Transcript levels were normalized to *GAPDH* and calculated using the 2^−ΔΔCt^ method. Data are presented as the mean ± SD of three independent biological replicates. Differences between AKAV-infected cells and the corresponding time-matched mock controls were analyzed by two-way ANOVA followed by Sidak’s multiple-comparison test. ns, not significant; * *p* < 0.05, ** *p* < 0.01, and *** *p* < 0.001.

**Figure 5:**
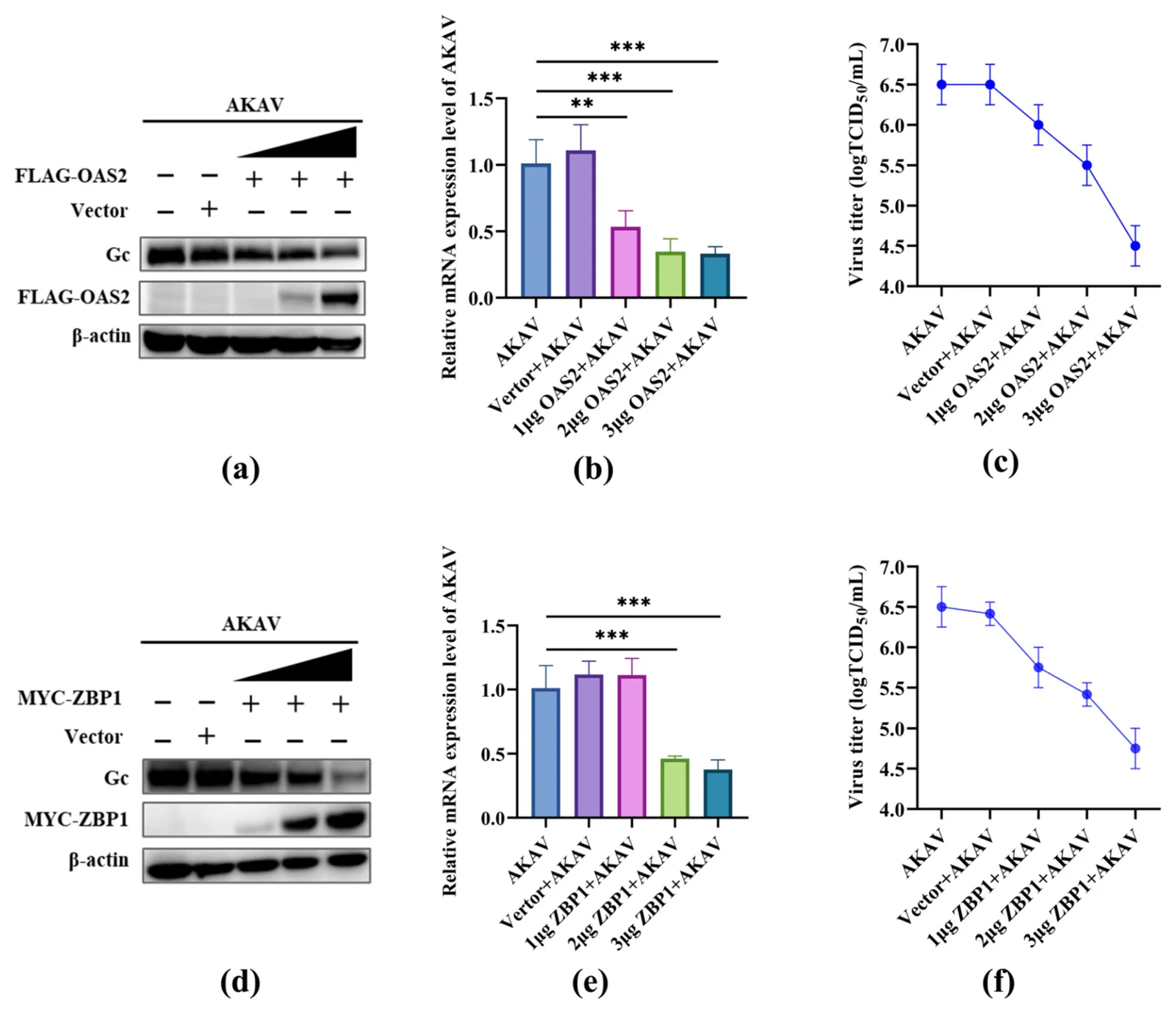
Effects of OAS2 and ZBP1 overexpression on AKAV replication. BHK-21 cells were transfected with 1, 2, or 3 μg of plasmids expressing FLAG-tagged OAS2 (**a**–**c**) or Myc-tagged ZBP1 (**d**–**f**) and subsequently infected with AKAV. AKAV-infected cells without plasmid transfection and cells transfected with the corresponding empty vector before infection were included as controls. Tagged protein expression and AKAV Gc protein abundance were examined by Western blotting, with β-actin as the loading control (**a**,**d**). Relative AKAV RNA levels were measured by RT-qPCR (**b**,**e**), and infectious virus titers in the culture supernatants were determined by TCID_50_ assay (**c**,**f**). Data are presented as the mean ± SD of three independent experiments. Statistical comparisons were performed using one-way ANOVA followed by Dunnett’s multiple-comparison test, with AKAV-infected cells without plasmid transfection as the reference group. ** *p* < 0.01, and *** *p* < 0.001 (the original Western blotting pictures can be found in File S1).

**Table 1:** Primer sequences used for RT-qPCR.

Primer Name	Primer Sequence (5′→3′)
BST2-F	ACGAACACCGAGGAGGACATAAG
BST2-R	CCAGCAGGAGCAGCAGGAC
ZBP1-F	AGAGGATTCTGGAGGTGTTGAGG
ZBP1-R	TCTTGTGGAGGAGCTGGTTGAG
ARHGEF6-F	AAGAGATTCCTCCGTAGTTCAGACC
ARHGEF6-R	TATAAGGTGTGACCGCCGAGTG
OAS2-F	CTTGTCGTCTTCCTCACCAATCTC
OAS2-R	TGTCTTTGATAGGCTTCCAACTGTC
IFIT1-F	GCCACCCTCACCCAGTACC
IFIT1-R	GCTCACATCGTCCACCACATC
IFNβ1-F	TGTCAATATCTGCCATCGCCAATG
IFNβ1-R	TGTCCTTCTCCCTCAGCTCTATG
AKAV-NSs-F	TAAGACGCCACAACCAAGTGT
AKAV-NSs-R	CCGAAATGCGATGGAGCGTA
GAPDH-F	TGAATACGGCTACAGCAACA
GAPDH-R	AGGCCCCTCCTGTTATTATG

**Table 2:** Read quality and alignment performance of the RNA-seq samples.

Sample	Clean Reads	Non-rRNA Reads (%)	Q30 (%)	GC Content (%)	Uniquely Mapped Reads (%)	Total Mapped Reads (%)
MOCK-12h-1	47,165,394	99.42	94.34	47.84	95.48	97.34
MOCK-12h-2	45,051,198	99.49	94.69	47.87	95.40	97.26
MOCK-12h-3	41,481,478	99.32	94.87	47.71	95.50	97.40
AKAV-12h-1	40,814,306	99.35	94.53	48.01	95.25	97.14
AKAV-12h-2	41,251,038	99.45	94.07	47.88	95.09	96.94
AKAV-12h-3	37,473,674	99.44	93.71	47.53	95.25	97.11
MOCK-24h-1	38,797,122	99.51	94.12	47.73	95.27	97.10
MOCK-24h-2	43,170,512	99.50	94.54	48.01	95.27	97.15
MOCK-24h-3	40,489,744	99.46	94.55	47.98	95.25	97.13
AKAV-24h-1	44,892,642	99.44	94.49	47.97	95.26	97.21
AKAV-24h-2	43,911,774	99.44	94.24	48.09	95.27	97.22
AKAV-24h-3	43,435,876	99.45	94.23	48.11	94.95	96.88

## Data Availability

The original contributions presented in the study are included in the article/Supplementary Materials. Further inquiries can be directed to the corresponding authors.

## Supplementary Materials

The following supporting information can be downloaded at https://www.mdpi.com/article/10.3390/vetsci13101015/s1, Table S1: Differentially expressed genes in AKAV-infected versus mock-infected MDBK cells at 12 h post-infection; Table S2: Differentially expressed genes in AKAV-infected versus mock-infected MDBK cells at 24 h post-infection; File S1: Original images of Western-blotting.
